# Outcomes of proton therapy for non-small cell lung cancer in patients with interstitial pneumonia

**DOI:** 10.1186/s13014-022-02027-0

**Published:** 2022-03-21

**Authors:** Shingo Hashimoto, Hiromitsu Iwata, Yukiko Hattori, Koichiro Nakajima, Kento Nomura, Kensuke Hayashi, Toshiyuki Toshito, Eiko Yamamori, Kenji Akita, Jun-etsu Mizoe, Hiroyuki Ogino, Yuta Shibamoto

**Affiliations:** 1 Department of Radiology, Nagoya City University Graduate School of Medical Sciences, 1-Kawasumi, Mizuho-cho, Mizuho-ku, Nagoya, 467-8601 Japan; 2 Department of Radiation Oncology, Nagoya Proton Therapy Center, Nagoya City University West Medical Center, Nagoya, Japan; 3 Department of Proton Therapy Technology, Nagoya Proton Therapy Center, Nagoya City University West Medical Center, Nagoya, Japan; 4 Department of Proton Therapy Physics, Nagoya Proton Therapy Center, Nagoya City University West Medical Center, Nagoya, Japan; 5 Department of Diagnostic Radiology, Tohoku University Hospital, Sendai, Japan; 6 Department of Respiratory Medicine, Thoracic Oncology Center, Nagoya City University West Medical Center, Nagoya, Japan; 7 Sapporo High Functioning Radiotherapy Center, Hokkaido Ohno Memorial Hospital, Sapporo, Japan; 8 Narita Memorial Proton Center, Toyohashi, Japan

**Keywords:** Proton therapy, Interstitial pneumonia, Idiopathic pulmonary fibrosis, Lung cancer, Radiation pneumonitis, Quality of life

## Abstract

**Background:**

Interstitial pneumonia (IP) is a disease with a poor prognosis. In addition, IP patients are more likely to develop lung cancer. Since IP patients frequently develop toxicities during cancer treatment, minimally invasive cancer treatment is warranted for such patients to maintain their quality of life. This study retrospectively investigated the efficacy and safety of proton therapy (PT) for non-small cell lung cancer (NSCLC) in patients with IP.

**Methods:**

Twenty-nine NSCLC patients with IP were treated with PT between September 2013 and December 2019. The patients had stage IA to IIIB primary NSCLC. Ten of the 29 patients exhibited the usual interstitial pneumonia pattern. The prescribed dose was 66–74 Grays (relative biological effectiveness) in 10–37 fractions.

**Results:**

The median follow-up period was 21.1 months [interquartile range (IQR), 15.6–37.3] for all patients and 37.2 months (IQR, 24.0–49.9) for living patients. The median patient age was 77 years (IQR, 71–81). The median planning target volume was 112.0 ml (IQR, 56.1–246.3). The 2-year local control, progression-free survival, and overall survival rates were 85% (95% confidence interval: 57–95), 30% (15–47), and 45% (26–62), respectively. According to the Common Terminology Criteria for Adverse Events (version 4.0), grade 3 acute radiation pneumonitis (RP) was observed in 1 patient. Two patients developed grade 3 late RP, but no other patients experienced serious toxicities. The patients’ quality of life (European Organization for Research and Treatment of Cancer QLQ-C30 and QLQ-LC13 and SF-36) scores had not changed after 3 months.

**Conclusions:**

﻿PT may be a relatively safe treatment for NSCLC patients with IP, without deteriorating quality of life scores within 3 months.

## Background

Interstitial pneumonia (IP) is a group of diffuse parenchymal lung disorders that can affect mortality [1]. The classification of IP is based on pathological and imaging findings. Among the various types of IP, idiopathic pulmonary fibrosis (IPF) is associated with the worst prognosis [2]. With an estimated incidence of 4.6–16.3 per 100,000, IPF is the most common form of idiopathic IP [3]. Although the course of the disease is variable and unpredictable, the median survival time from diagnosis is 2–4 years [4]. IP, especially IPF, is often accompanied by lung cancer (frequency: 10–20% of cases) [5].

Systemic therapy for non-small cell lung cancer (NSCLC) has changed markedly over the last 15 years. However, most clinical trials exclude lung cancer patients with IP; and hence, their treatment has not improved. This is because surgery, drug therapy, and radiotherapy can occasionally lead to the fatal exacerbation of IP [6–8]. Lung cancer treatment in patients with IP requires the prognoses of both the lung cancer and IP to be estimated and compared. If the prognosis of the lung cancer is considered to be worse than that of the IP, the safest treatment from among surgery, drug therapy, and radiotherapy is selected, taking the patient’s condition into account.

Radiotherapy using photon beams, including conventional radiotherapy and stereotactic body radiotherapy (SBRT), has been reported to be difficult in IPF patients due to the high incidence of life-threatening pneumonia seen after treatment [8, 9]. On the other hand, proton therapy (PT) is gaining attention as a new and effective treatment option. The greatest advantage of PT is that the physical properties of proton beams, especially with respect to the Bragg peak, improve the dose distribution; i.e., PT reduces unnecessary doses to multiple sensitive organs at risk (OAR) and enables high-dose, uniform irradiation of tumors [10].

In recent years, many PT facilities have been built, and the number of lung cancer patients receiving PT is increasing. In Japan, medical insurance coverage of PT for NSCLC is currently under active debate, and the government is requesting further evidence. Although the outcomes of PT are gradually being revealed by numerous investigations, there are still few reports about PT for lung cancer patients with IP. The purpose of this study was to evaluate the incidence of post-PT adverse events, especially in the lungs, in NSCLC patients with IP. We also evaluated health-related quality of life (HRQOL), an important outcome measure used in clinical trials, before and after PT.

## Methods

### Study design

**Fig. 1:**
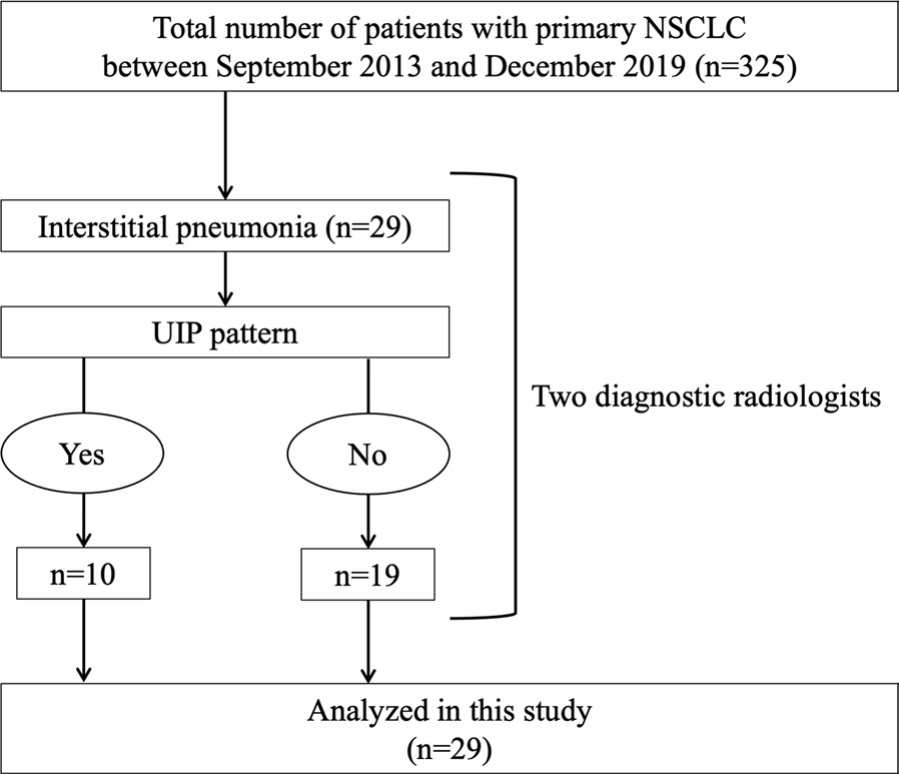
Study flow diagram. Two diagnostic radiologists diagnosed interstitial pneumonia and presence of UIP pattern based on high resolution CT images before PT

We retrospectively analyzed the outcomes and safety of PT for NSCLC patients with IP treated in previous and ongoing prospective clinical studies of PT. This study was approved by the institutional review board of Nagoya City Hospital (numbers 20-04-327-07). Written informed consent was obtained from all subjects. Between September 2013 and December 2019, 325 patients were enrolled in prospective studies at Nagoya Proton Therapy Center. Two diagnostic radiologists diagnosed IP based on high-resolution computed tomography (CT) images obtained before the PT. Twenty-nine patients with IP were extracted from among the 325 patients and evaluated in this study. A flow chart for the patient selection is shown in Fig. 1. The patients’ CT images were also examined in detail to determine the presence/absence of the usual interstitial pneumonia (UIP) pattern, which is a clinical indicator of IPF. The radiographic diagnosis of the UIP pattern was based on bilateral, predominantly basal, predominantly subpleural, reticular abnormalities and ﻿honeycombing with or without traction bronchiectasis [11].

### Patient eligibility and disease staging

The inclusion criteria were as follows: (1) histologically confirmed NSCLC; (2) clinical stage IA to IIIC disease (8th edition of the TNM staging classification of the Union for International Cancer Control, UICC); (3) IP that was diagnosed based on high-resolution CT imaging with 1-mm slice thickness before the PT; (4) an Eastern Cooperative Oncology Group performance status of 0–2; (5) none of the OAR dose constraints being exceeded; (6) no previous irradiation of the target region for the PT; (7) no history of chemotherapy; (8) an age of ≥ 20 years; and (9) written informed consent provided.

The exclusion criteria were as follows: (1) pregnancy; (2) synchronous or metachronous cancer within the past 5 years; (3) active infectious disease; (4) other severe comorbidities, e.g., hypertension or diabetes mellitus; and (5) a severe psychological disorder. Medical inoperability and the suitability of the patients for chemotherapy were determined by multidisciplinary thoracic specialists, including thoracic surgeons and pulmonologists. Staging was performed based on magnetic resonance imaging (MRI) of the brain, CT of the chest and upper abdomen, and ^18^F-deoxyglucose-positron emission tomography-CT (PET-CT) within 1 month before the start of the PT. The diagnostic criteria of the UIP pattern on high-resolution CT imaging were as follows: (1) subpleural distribution with a basal predominance; and (2) honeycombing with or without peripheral traction bronchiectasis or bronchiolectasis. Patients with the UIP pattern on CT imaging were clinically diagnosed with IPF [12].

### PT and treatment planning

Our PT procedures were described in detail previously [13, 14]. PT was planned using the VQA planning system (version 3.0.5, Hitachi, Ltd., Tokyo, Japan) with the pencil-beam algorithm and was performed using the PROBEAT-III system (Hitachi, Ltd.) [15–17]. In patients that did not have lymph node metastasis, the prescribed isocenter dose was 66 Gy (relative biological effectiveness, RBE) in 10 fractions for peripherally located tumors and 72.6 GyRBE in 22 fractions for centrally located tumors. In cases involving lymph node metastasis, the isocenter dose was 70.2 GyRBE in 26 fractions for patients that did not receive chemotherapy and 70–74 GyRBE in 35–37 fractions to the primary site and 66 GyRBE in 33 fractions to the lymph nodes in patients that received chemotherapy. This resulted in biologically effective doses (calculated with an α/β ratio of 10 Gy) of 110, 97, 89, 84–89, and 79 GyRBE, respectively. All PT was performed once a day, 5 days a week. Two to four beam portals were used for each treatment. An RBE value of 1.1 was used based on International Commission on Radiation Units and Measurements (ICRU) Report 78 [18] and our previous investigation [19]. Based on the accumulated evidences of PT [20–26], the above-mentioned dose-fractionation schedules were determined by the Advanced Medical Council of the Ministry of Health, Labor and Welfare of Japan, and every institution was requested to adopt the schedules.

Patients with highly movable tumors underwent fiducial marker placement. When the tumor was located near a bronchus, ﻿three 1.5-mm gold markers were implanted using bronchoscopy. For tumors located away from the bronchi, 0.28- or 0.5-mm markers were percutaneously implanted according to the procedure reported for liver tumors [27]. Marker insertion was performed without serious pneumothorax occurring. Patients were immobilized in the supine position with our own device-free compressed shell fixation method to reduce the respiratory movement of the tumors [28]. CT simulations based on 4-dimensional CT (slice thickness: 2 mm), which was performed using a 16-row multi-detector CT scanner, were conducted for all patients. The planning target volume (PTV), dose constraints for normal tissues, and respiratory gating irradiation were described in detail previously [13, 14].

### Evaluation and follow-up

The patients were followed up at 6-week intervals until 6 months after the PT and at 3-month intervals thereafter. The routine follow-up studies included chest and upper abdominal CT scans and tumor marker examinations. MRI and PET-CT were usually performed annually or whenever necessary. Acute and late treatment-related toxicities, including radiation pneumonitis (RP), were assessed using the National Cancer Institute Common Toxicity Criteria for Adverse Events (version 4.0). The RP grades, excluding infection, were as follows: grade 1, asymptomatic (radiographic findings only); grade 2, radiographic findings plus symptomatic and not interfering with activities of daily living; grade 3, radiographic findings plus symptomatic and interfering with activities of daily living or O_2_ indicated; grade 4, radiographic findings plus life-threatening (ventilatory support indicated), and grade 5, radiographic findings plus death. Infectious pneumonitis was defined as pneumonitis that proved to be a bacterial infection by germ culture. Local recurrence was diagnosed based on the expansion of a consolidated fibrotic mass within the irradiated area on CT images and PET-CT. If recurrence was strongly suspected, a biopsy was performed, depending on the condition of the patient’s lungs. The response after PT was evaluated using the Response Evaluation Criteria in Solid Tumors (RECIST) [29]. HRQOL scores were calculated using the European Organization for Research and Treatment of Cancer (EORTC) core quality of life questionnaire (QLQ-C30, version 3.0), the EORTC quality of life questionnaire—lung cancer module (QLQ-LC13), and the Short-Form Health Survey (SF-36) before and 3 months after the PT [30–32].

### Statistical analysis

Local control (LC), progression-free survival (PFS), and overall survival (OS) rates were calculated using the Kaplan–Meier method from the date of the first round of the PT. The following dosimetric factors were examined with the use of dose-volume histograms of the lungs without the gross tumor volume (GTV): the mean lung dose (MLD), the lung volumes receiving doses of ≥ 5/10/20/40 GyRBE (the lung V_5GyRBE_, lung V_10GyRBE_, lung V_20GyRBE_, and lung V_40GyRBE,_ respectively). ﻿In addition, the conformity index (CI) was defined as the ratio of the volume receiving at least 95% of the prescribed dose to the PTV. A CI approaching 1 indicates better dose convergence. The homogeneity index (HI) (D2–D98%/D50%) of each plan was determined. An HI approaching 0 indicates better dose uniformity. These parameters were defined as outlined in ICRU Report 83 [33]. Dosimetric parameters and HRQOL scores were analyzed using the Mann–Whitney *U* test. *P* values of < 0.05 were considered to be significant. All statistical analyses were performed with EZR (version 1.51) [34].

## Results

### Representative case

**Fig. 2:**
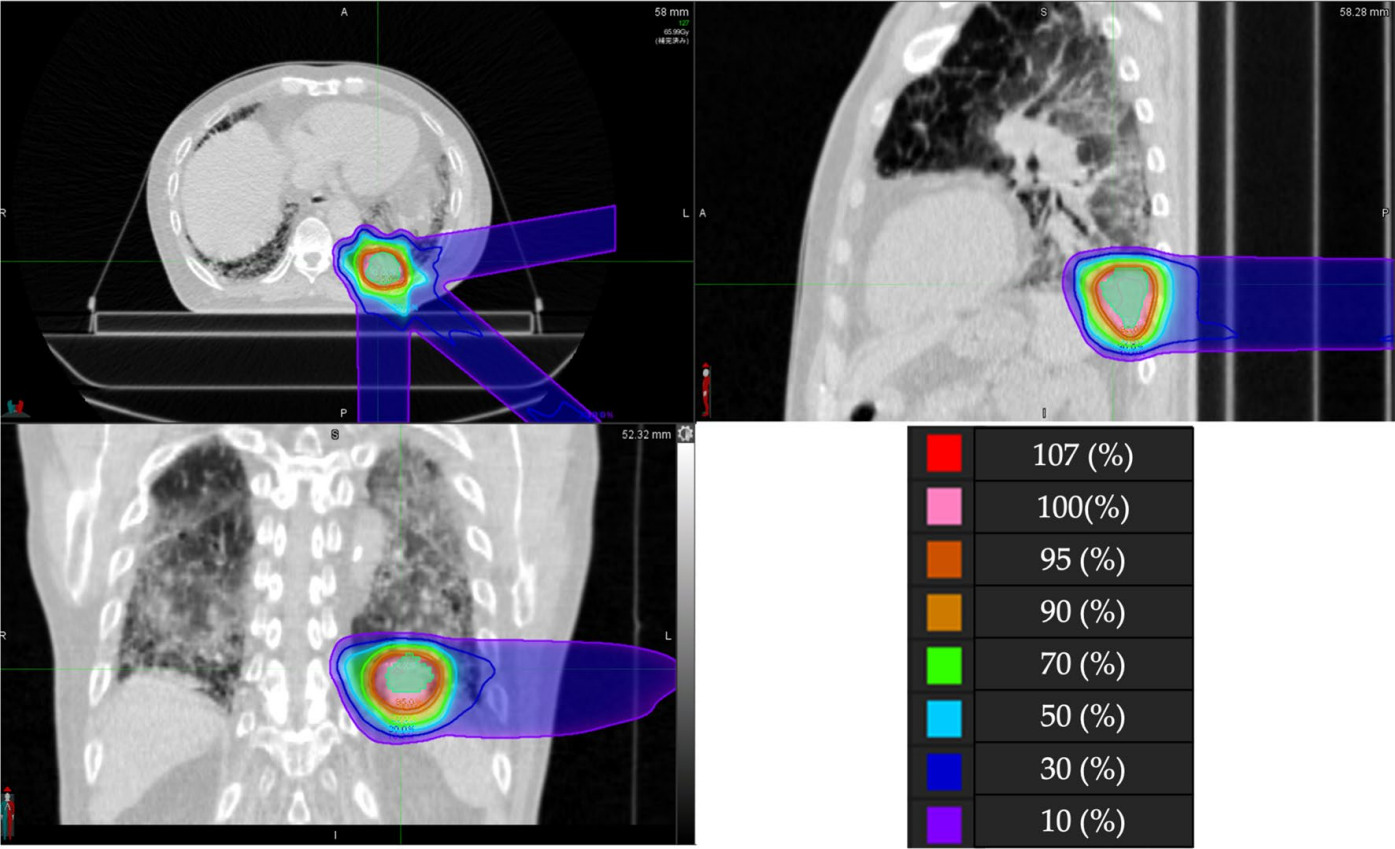
PT dose distribution map for a lung cancer patient with IPF. The area outside the outermost line received less than 10% of the prescribed isocenter dose of radiation. The planning target volume is shown in green

The PT plan for a representative patient is shown in Fig. 2. This 73-year-old male patient was diagnosed with IPF and received home oxygen therapy before lung cancer was found. A lung nodule had grown in the left lower lobe over time, but performing a biopsy was difficult because of the presence of IPF. After consulting our cancer board, the nodule was treated with PT under a diagnosis of cT2bN0M0 stage IIA lung cancer. The prescribed dose was 66 GyRBE in 6.6-GyRBE daily fractions. No serious toxicities developed during or after the treatment.

### Patients

The characteristics of the patients and tumors are summarized in Table 1. The UIP pattern was found in the lung fields of 10 patients. The median serum KL-6 level, serum surfactant protein D level, % vital capacity, and arterial O_2_ pressure level before the PT were 725 U/ml (IQR, 444–1200), 167.8 ng/ml (IQR, 96.1–257.9), 83.1% (IQR, 73.5–97.7), and 81.4 mmHg (IQR, 68.1–90.1), respectively. Eight patients underwent concurrent chemoradiotherapy and adjuvant chemotherapy. Three of them were treated with cisplatin and S-1, as described previously [14]. According to the National Comprehensive Cancer Network guidelines for NSCLC, four patients were treated with carboplatin, and the remaining patient was treated with docetaxel hydrate [35].

**Table 1:** Patient and tumor characteristics

Characteristics	Patients
Age (years)	
Median (IQR)	77 (71–81)
Sex	
Male/female	25/4
Performance status	
0/1/2	21/6/2
UICC (8th ed.) stage	
IA2/IA3/IB/IIA/IIB/IIIA/IIIB	5/2/3/1/5/5/8
UIP pattern	
Yes/no	10/19
Smoking	
Yes/no	24/5
Histopathology	
Squamous cell carcinoma	13
Adenocarcinoma	9
Large cell carcinoma	1
NSCLC	2
Clinical malignancy	4

*IQR* interquartile range, *UICC (8th ed.)* union for international cancer control 8th edition, *UIP* usual interstitial pneumonia, *NSCLC* unclassified non-small cell lung cancer

### Survival and local control

**Fig. 3:**
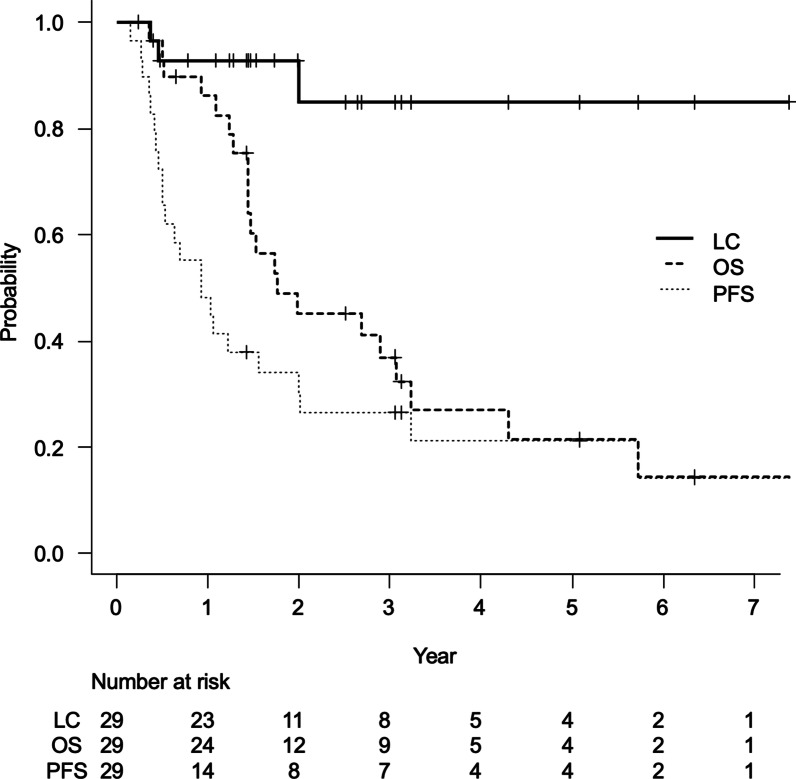
Curves for local control (LC) (solid line), overall survival (OS) (dotted line) and progression- free survival (PFS) (broken line) for all patients

At the time of the analysis, 7 patients were alive, and 22 patients had died. The median duration of the follow-up period was 21.1 months (IQR, 15.6–37.3) for all patients and 37.2 months (IQR, 24.0–49.9) for the living patients. Local recurrence occurred in 3 patients. One had T3N0M0 stage IIB NSCLC with the UIP pattern, and the other two had T2bN1M0 stage IIB and T3N2M0 stage IIIB NSCLC, respectively, without the UIP pattern. Nineteen (65%) lesions exhibited a complete (17%) or partial response (48%). Seven (24%) lesions were classified as stable disease. Regional lymph node recurrence was observed in 11 patients, and distant metastasis was seen in 11 patients. The rates of LC, PFS, and OS at 2 years were 85% (95% confidence interval: 57–95), 30% (15–47), and 45% (26–62), respectively (Fig. 3). The median survival time was 1.8 years.

### Toxicities

Grade 3 acute radiation pneumonitis was observed in one patient (3%) at 3 months after the PT. Two patients (7%) developed late grade 2 RP and received steroids. Two patients (7%) experienced grade 3 late RP and required home oxygen therapy. The overall incidence of grade 2 or 3 late RP was 5% in patients without the UIP, but 30% in patients with the UIP (Table 2). A grade 2 rib fracture occurred in one patient (3%). No cases of symptomatic dermatitis were observed. In addition, there were no other grade 3 or worse toxicities during either the acute or late observation period.

**Table 2:** Incidence of late radiation pneumonitis

	Grade 0/1	Grade 2	Grade 3
UIP ( +)	7	2	1
UIP ( −)	18	0	1

*UIP* usual interstitial pneumonia

### Dosimetric analyses

Table 3 shows treatment characteristics. The PTV D_50%_ (the dose received by 50% of the volume of the PTV) values were all around 100%, and the PTV D_98%_ values (which are nearly equal to D_max_ according to ICRU Report 83 [33]) were ≤ 105%. The PTV median D_95%_ and D_98%_ (which are nearly equal to D_min_ according to ICRU Report 83 [33]) values were 94.7% and 90.2%, respectively, while the first-quartile D_95%_ and D_98%_ values were 85.8% and 79.9%, respectively. This means that target coverage was sacrificed to protect the lungs in some cases. The median CI was 1.34 (IQR, 0.87–1.48), and the median HI was 0.13 (0.09–0.23).

**Table 3:** Treatment characteristics

Characteristics	Median	IQR
*PTV*		
Volume (ml)	112.0	(56.1–246.3)
D_2%_ (%)	102.7	(101.9–103.8)
D_50%_ (%)	100.1	(98.7–100.5)
D_95%_ (%)	94.7	(85.8–97.4)
D_98%_ (%)	90.2	(79.9–94.1)
CI	1.34	(0.87–1.48)
HI	0.13	(0.09–0.23)
*Lung-GTV*		
MLD (GyRBE)	7.9	(3.4–11.6)
V_5GyRBE_ (%)	22.2	(11.1–31.3)
V_10GyRBE_ (%)	19.5	(8.8–27.3)
V_20GyRBE_ (%)	15.8	(6.4–20.7)
V_40GyRBE_ (%)	8.0	(3.8–13.3)

*IQR* interquartile range, *PTV* planning target volume, *D*_*2%*_*–D*_*98%*_ the dose received by x% of the volume of the PTV, *CI* conformity index, *HI* homogeneity index, *GTV* gross tumor volume, *MLD* mean lung dose, *RBE* relative biological effectiveness, *V*_*5GyRBE*_*,V*_*10GyRBE*_*,V*_*20GyRBE*_*, and V*_*40GyRBE*_ volume receiving a dose of ≥ 5/10/20/40 GyRBE

**Fig. 4:**
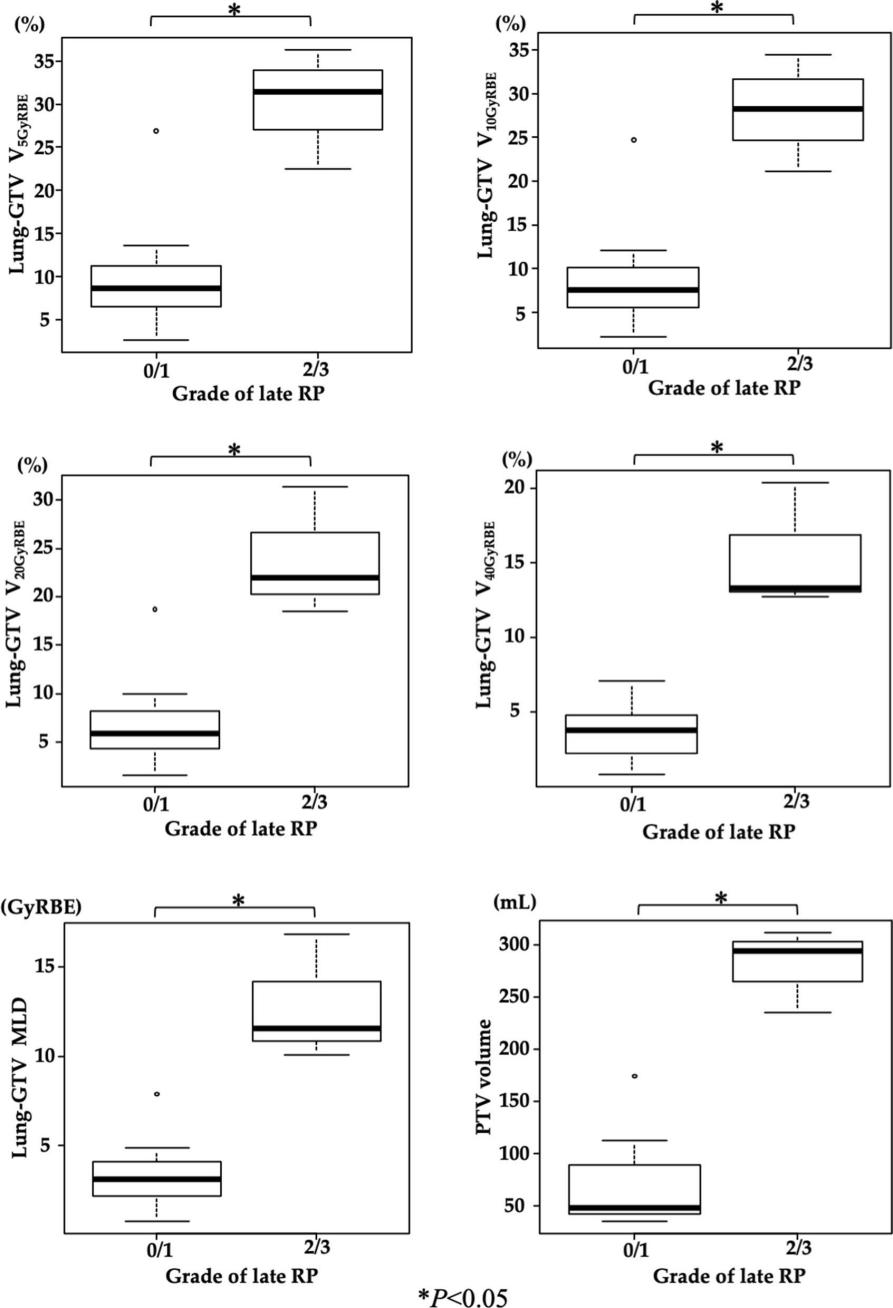
Dosimetric analyses of patients with the UIP (N = 10, Mann–Whitney *U* test). Stars indicate outliers

The dosimetric parameters for the lungs without the GTV did not significantly affect the incidence of grade ≥ 2 late (Table 4). Therefore, we narrowed down the analysis to the 10 patients that exhibited the UIP pattern (Fig. 4). The V_5GyRBE_, V_10GyRBE_, V_20GyRBE_, V_40GyRBE_, and MLD values of the lungs without the GTV were significantly higher in the group with grade ≥ 2 late RP. In addition, the PTV was significantly larger in the group with grade ≥ 2 late RP.

**Table 4:** Relationships between dose-volume-histogram parameters and the incidence of grade 2 or higher late radiation pneumonitis

	Grade ≤ 1		Grade ≥ 2		*P* value
	Pneumonitis (N = 25)		Pneumonitis (N = 4)		
	Median	IQR	Median	IQR	
*Lung-GTV*					
MLD (GyRBE)	7.0	(3.4–11.2)	10.8	(8.2–12.9)	0.36
V_5GyRBE_ (%)	21.8	(11.1–28.0)	26.7	(19.0–32.7)	0.44
V_10GyRBE_ (%)	18.6	(8.8–25.7)	24.7	(17.7–29.9)	0.44
V_20GyRBE_ (%)	13.7	(6.4–19.6)	20.2	(15.1–24.3)	0.37

*IQR* interquartile range, *GTV* gross tumor volume, *MLD* mean lung dose, *RBE* relative biological effectiveness, *V*_*5GyRBE*_*, V*_*10GyRBE*_*, V*_*20GyRBE*_*, and V*_*40GyRBE*_volume receiving a dose of ≥ 5/10/20/40 GyRBE

### Quality of life

The changes in the EORTC QLQ-C30, EORTC QLQ-LC13, and SF-36 scores seen at 3 months after the PT are shown in Table 5. There were no significant changes in any of the HRQOL scores during the 3-month follow-up period. High scores on the QLQ-C30 functional scales and low scores on the QLQ-C30 symptom scales and QLQ-LC13 are rated as good. The scores for fatigue and dyspnea on the QLQ-C30 and dyspnea and coughing on the OLQ-LC13 were markedly worse than those for the other items. Before the PT, the SF-36 subscale scores were all ≥ 50, except for the general health subscale score. Before treatment, the three-physical component summary, three-role-social component summary, and two-physical component summary scores were ≤ 50, which were lower than the national standard values. However, these scores did not decrease significantly after the PT.

**Table 5:** Changes in quality-of-life scores after proton therapy

Variable	Pre-PT	3 Months	*P* value
	(N = 28)	(N = 22)	
*EORTC QLQ-C30*			
Global health status/QOL	50.9 (27.8)	49.6 (27.0)	0.95
*Functional scales*			
Physical functioning	73.8 (21.9)	75.8 (17.8)	0.98
Role functioning	71.4 (27.5)	77.3 (19.6)	0.58
Emotional functioning	79.5 (19.3)	89.0 (13.0)	0.07
Cognitive functioning	74.4 (24.6)	78.0 (20.8)	0.72
Social functioning	80.9 (20.6)	89.4 (15.9)	0.12
*Symptom scales/items*			
Fatigue	34.5 (24.8)	29.3 (17.7)	0.53
Nausea and vomiting	2.98 (9.13)	3.03 (11.1)	0.88
Pain	12.5 (20.6)	6.82 (11.1)	0.46
Dyspnea	35.7 (25.6)	45.4 (22.0)	0.19
Insomnia	17.9 (23.1)	18.2 (24.6)	0.98
Appetite loss	15.5 (26.4)	10.6 (23.9)	0.45
Constipation	19.1 (27.9)	19.7 (24.5)	0.75
Diarrhea	8.33 (17.3)	9.08 (15.2)	0.70
Financial difficulties	26.2 (33.2)	15.2 (24.6)	0.21
*EORTC QLQ-LC13*			
Symptom scales/items			
Dyspnea	27.4 (21.7)	31.8 (16.2)	0.32
Coughing	30.9 (22.1)	36.4 (30.7)	0.51
Hemoptysis	3.57 (10.5)	4.54 (11.7)	0.77
Sore mouth	4.76 (11.9)	3.03 (9.80)	0.59
Dysphagia	7.14 (21.0)	4.55 (15.6)	0.60
Peripheral neuropathy	8.33 (14.7)	12.1 (19.4)	0.55
Alopecia	13.1 (27.7)	3.03 (9.80)	0.14
Pain in chest	7.14 (16.6)	10.6 (15.9)	0.30
Pain in arm or shoulder	9.52 (20.0)	10.6 (18.9)	0.70
Pain in other parts	11.9 (14.7)	9.09 (18.3)	0.82
*SF-36*			
Symptom scales/items			
Physical functioning	66.4 (23.8)	68.0 (21.3)	1.00
Role-physical	57.8 (33.0)	65.1 (26.6)	0.40
Bodily pain	75.9 (27.5)	80.2 (19.8)	0.86
General health	48.6 (16.1)	50.5 (18.2)	0.35
Vitality	58.5 (25.2)	61.1 (22.6)	0.70
Social functioning	70.5 (29.7)	77.8 (20.4)	0.55
Role-emotional	64.3 (31.4)	70.5 (29.5)	0.50
Mental health	66.1 (22.0)	67.3 (19.4)	0.73
3-Physical CS*	40.6 (11.1)	41.3 (10.5)	0.95
3-Mental CS*	55.6 (10.2)	55.8 (8.29)	0.95
3-Role-social CS*	39.9 (15.1)	43.5 (10.9)	0.43
2-Physical CS*	35.3 (14.3)	38.4 (12.6)	0.54
2-Mental CS*	53.7 (10.7)	54.5 (8.54)	0.82

*Scored using factor coefficients based on the 1995 Japan National Survey; *CS* component summary; Data are shown as mean (standard deviation) values

## Discussion

In the present study, grade 3 acute RP only occurred in one patient (3%). The incidence of grade 2 or 3 late RP after PT was 35%, while there were no cases of grade 4 or 5 late RP. Table 6 summarizes the frequencies of grade 3, 4, or 5 late RP in IP patients in previous studies. Lee et al. [8] reported that 3D-CRT produced grade 3 RP in 40% of the patients and grade 4 or 5 RP in 33%. According to Yamashita et al. [9], grade 4 or 5 RP developed in 54% of the patients after SBRT. On the other hand, Tsurugai et al. [36] reported that SBRT produced grade 3 RP in only 9.5% and grade 4 or 5 RP in 2.4%. It seems difficult to explain the large difference in the incidence of grade ≥ 3 RP after SBRT, but it may be partly related to the dose prescription method; Yamashita et al. [9] prescribed the dose to the PTV isocenter, while Tsurugai et al. [36] prescribed the dose to the 80% or 60% isodose line of the maximal dose. The latter method may reduce the lung dose. Using PT, Ono et al. [37] reported an incidence of 6.3% for grade 3 RP and 6.3% for grade 4 or 5 RP. In the present study of PT, there were only two cases (6.9%) of grade 3 late RP, even though the median PTV size was 112 ml compared to 45 ml in Tsurugai's study [36]. These studies are suggesting that PT is associated with a lower risk of fatal pneumonia among lung cancer patients with IP than X-ray therapy. This may be due to the physical characteristics of PT, as it reduces the doses delivered to the surrounding normal organs [10, 38].

**Table 6:** Previous studies of late radiation pneumonitis in patients with interstitial pneumonitis

References	Treatment	Number of patients	Median total dose (Gy)	Dose per fraction (Gy)	Pneumonitis	
					Grade 3	Grade 4/5
Lee et al. [8]	3D-CRT	15	56.9	1.8–2.0	6 (40%)	5 (33%)
Yamashita et al. [9]	SBRT	13	48	12	N/A	7 (54%)
Tsurugai et al. [36]	SBRT	42	40–60	8–22	4 (9.5%)	1 (2.4%)
Ono et al. [37]	PT	16	80 (RBE)	3.2 (RBE)	1 (6.3%)	1 (6.3%)
Present study	PT	29	66–74 (RBE)	6.6–2.0 (RBE)	2 (6.9%)	0 (0%)

*3D-CRT* 3D conformal radiotherapy, *SBRT* stereotactic body radiotherapy, *PT* proton therapy, *RBE* relative biological effectiveness, *N/A* not applicable

Previous studies have shown that IPF patients with lung cancer have shorter survival times than patients with IPF alone [39, 40]. However, many treatment-related deaths have been reported in lung cancer patients with IPF. Surgery, such as lobectomy and biopsies, also worsens IPF. The reported postoperative IPF exacerbation rates range from 9.3 to 30% [6, 41, 42]. The risk of pulmonary toxicity from drug therapy, such as pemetrexed, has been reported to be approximately 3.5% in patients without IP, 12.0% in patients with IP, and up to 16.7% in patients with IPF [43]. In our study, 10 patients that exhibited the UIP pattern, which is suggestive of IPF, were treated with PT, and only one of them developed late grade 3 RP. There were no deaths associated with PT. Therefore, PT can be considered to be relatively safe. However, even narrowly localized radiotherapy for patients with IPF was reported to lead to marked variation in the frequency of RP [44]. Therefore, the necessity of interventions, including PT, should be carefully assessed in lung cancer patients with IPF.

Conventional radiotherapy for lung cancer patients with IP may be associated with a high risk of life-threatening pneumonia. SBRT may be safer if patients that were at high risk were excluded based on pretreatment CT evaluations or the measurement of biomarker levels [9, 36]. However, SBRT is generally used as a treatment option for early stage lung cancer, and treating large targets with SBRT is technically difficult [45, 46]. In our study, the median PTV of the patients treated with PT was large due to the inclusion of stage I to III patients, while the doses delivered to the lungs were kept low (Table 3). The lower lobe lesion shown in Fig. 2 might have been controlled with X-ray therapy, but this patient had active IPF. In X-ray therapy, low- and medium-dose volumes (V20Gy and V5Gy) tend to spread in the surrounding organs, which may result in a higher risk of RP. A correlation has been reported between RP and irradiation dose to the lungs [47, 48]. Owing to the physical properties of PT, undesirable irradiation of the lungs can be reduced compared to X-ray therapy. Although the stages and doses were various in our study, our data may be useful in evaluating toxicity in relation to the dose. Our study suggests that the physical properties of PT are advantageous. Especially in stage III lung cancer patient with IP, PT may be a safer treatment, considering the increased risk of RP due to the larger treatment volume.

QOL evaluations are important for comparing treatment modalities. Surgery is highly invasive and often leads to poor QOL. In a previous study, it was reported that patients’ QLQ-C30 scores had not returned to their preoperative levels at 6 months after lung cancer surgery [49]. Postoperative patients tend to experience persistent physical function problems, such as shortness of breath and pain in the arms and chest [50]. Reductions of 10% in the physical and mental component summary scores of the SF-36 from the baseline after lung cancer surgery have been reported to be associated with a high risk of death [51]. Although there is no consensus on what constitutes a significant difference in QOL data, a 10% difference in the SF-36 summary score is generally considered to be a clinically relevant difference. In our study, no significant reductions in HRQOL scores were seen after PT. As this study focused on lung cancer patients with IP, PT can be considered to be a less invasive treatment. However, the changes in QLQ-LC13 dyspnea scores seen at 3 months after radiotherapy have been shown to be correlated with lung V_30Gy_, V_40Gy_, V_50Gy_, and MLD values [52]. Previous studies have suggested that a lung V_40Gy_ cut-off value of 11% exhibits good sensitivity and specificity as a predictor of dyspnea. Our results showed that grade 2 or 3 late pneumonia developed in patients with lung V_40GyRBE_ values of > 11% (Table 4). The indications for PT for large PTV that require wide-field irradiation must be carefully judged in consideration of the risks and benefits.

This study had several limitations. First, Dosimetric analyses of the PTV showed that the D_50%_ tended to be relatively well preserved, but D_95%_ was sacrificed in some cases to ensure lung safety (Table 3). Sacrificing the PTV D_95%_ in this manner may be clinically acceptable, but it may negatively affect long-term prognosis. We try to achieve both high PTV coverage and low lung exposure using respiratory-gated irradiation with gold marker implantation [5]. Second, the HRQOL survey period was only 3 months. This was because in our prospective clinical studies the HRQOL surveys were scheduled to be conducted at 3 and 24 months after the PT. However, at 24 months sufficient data were not available for some patients due to the length of the follow-up period being too short or an HRQOL survey not being performed. Further case accumulation and multicenter trials will be needed to assess late toxicities. Third, patient selection bias must also be considered. Only patients who were judged to be suitable for this costly treatment by a pulmonologist were referred to our facility. In Japan, PT for lung cancer is not covered by medical insurance, and only wealthy people can receive this treatment. Thus, the prognosis of the patients in our study may have been abnormally good, as the patients probably had access to adequate standard medical support in addition to PT. Finally, patients with heterogeneous stages were enrolled in our study, and 22 of the 29 patients were deceased at the time of analysis. Patients with advanced disease may have died from lung cancer-related events before the occurrence of proton therapy-related events. Our study included only 29 patients with a median follow-up period of 21.1 months. However, RP is usually observed by 6 months after irradiation [53], and all patients have been observed beyond that period; thus the safety of PT was suggested. On the other hand, a recent systematic review did not suggest the superiority of PT over photon therapy for early-stage lung cancer patients with concomitant IP [54]. Analysis of the SBRT dose parameters in the article revealed that V_20Gy_ ≤ 6.5% and MLD ≤ 4.5 Gy were associated with lower mortality [54]. To clarify the safety of PT, excluding the above-mentioned bias, future studies should include only patients with early-stage lung cancer complicated by IP. PT may reduce mortality from adverse events because its physical properties make it easier to suppress V_20Gy_ and MLD.

Immune checkpoint inhibitors have been developed in recent years, and many patients will continue to be treated with them. In a prospective study in which nivolumab was administered to 6 NSCLC patients with mild IP, no life-threatening pneumonia occurred [55]. Even when they are used in combination with radiotherapy, there are many uncertainties regarding the risk of immune checkpoint inhibitors in patients with IP. There are also reports suggesting that a history of thoracic radiation is a risk factor for pneumonia during treatment with immune checkpoint inhibitors [56]. Our study showed that PT could reduce the radiation dose delivered to normal lung tissue, and the incidence of clinically problematic pneumonia was low. When immune checkpoint inhibitors need to be given to lung cancer patients with IP, PT could be useful for reducing the risk of adverse events. Therefore, at our facility, several IP patients with stage III NSCLC have been treated with durvalumab as maintenance therapy after chemotherapy combined with PT after approval was granted by the cancer board. The results of a prospective trial of this approach will also be reported in the future. We hope that PT can contribute to safer treatment in many lung cancer patients with IP.

## Conclusions

PT appears to be a safer treatment for NSCLC in patients with IP than conventional radiotherapy and SBRT. QOL scores did not deteriorate within 3 months after PT. When patients that exhibit the UIP pattern require clinical treatment, PT may be considered as a treatment option.

## Data Availability

The data presented in this study are available on request from the corresponding author. The data are not publicly available due to institutional guidelines.

## Abbreviations

IP: Interstitial pneumonia
PT: Proton therapy
NSCLC: Non-small cell lung cancer
IQR: Interquartile range
RP: Radiation pneumonitis
IPF: Idiopathic pulmonary fibrosis
SBRT: Stereotactic body radiotherapy
OAR: Organ at risk
HRQOL: Health-related quality of life
CT: Computed tomography
UIP: Usual interstitial pneumonia
UICC: Union for international cancer control
MRI: Magnetic resonance imaging
PET-CT: Positron emission tomography-CT
RBE: Relative biological effectiveness
ICRU: International commission on radiation units and measurements
PTV: Planning target volume
RECIST: Response evaluation criteria in solid tumors
EORTC: European Organization for Research and Treatment of Cancer
QLQ-C30: The EORTC core quality of life questionnaire
QLQ-LC13: The EORTC quality of life questionnaire—lung cancer module
SF-36: The short-form health survey
LC: Local control
PFS: Progression-free survival
OS: Overall survival
GTV: Gross tumor volume
MLD: Mean lung dose
CI: Conformity index
HI: Homogeneity index
